# Supplementary material for: An enhanced spatial-temporal graph convolution network with high order features for skeleton-based action recognition
Source: PLoS One. 2025 Oct 9;20(10):e0332815. doi: 10.1371/journal.pone.0332815 (PMC12510661; doi:10.1371/journal.pone.0332815)
Supplement: Attachment — Submitted filename: Response to Reviewers.docx [file pone.0332815.s001.docx]

**Reviewer Response Report**

I would like to express my sincere gratitude to the Editor and the reviewer. Your valuable feedback and insights are greatly appreciated, and we are committed to addressing all your comments and suggestions to enhance the quality and impact of our work.

**Editor's Journal comments and responses**

- **Comment 1**: [*Please ensure that your manuscript meets PLOS ONE's style requirements, including those for file naming. The PLOS ONE style templates can be found at….*]

**Response** : We would like to inform you that we have adhered to PLOS ONE's style requirements as outlined in the PLOS ONE style templates.

- **Comment 2**: [*We suggest you thoroughly copyedit your manuscript for language usage, spelling, and grammar. If you do not know anyone who can help you do this, you may wish to consider employing a professional scientific editing service*.**]**
- **Response**: We sincerely thank the reviewers and the editorial office for their suggestion regarding the language quality of our manuscript. In response, we have carefully and thoroughly revised the manuscript to improve grammar, spelling, and overall readability. These revisions were performed by the authors and colleagues with strong proficiency in academic English. Please refer to the revised version of the manuscript for the updated content..
- **Comment 3**: [*Please note that PLOS ONE has specific guidelines on code sharing for submissions in which author-generated code underpins the findings in the manuscript. In these cases, all author-generated code must be made available without restrictions upon publication of the work.***]**

**Response** : Thank you for your concerns regarding the  PLOS ONE guidelines on code sharing requirement. We assure you that we are fully committed to transparency and are willing to provide any details about the source code to reviewers upon request.

- **Comment 4**: [*PLOS requires an ORCID iD for the corresponding author in Editorial Manager on papers submitted after December 6th, 2016*.**]**

**Response** : As per the journal’s requirements, I have created an ORCID iD for the corresponding author (0009-0003-4633-7530), which has also been updated in Editorial Manager.

- **Comment** 5: [ *If the reviewer comments include a recommendation to cite specific previously published works, please review and evaluate these publications to determine whether they are relevant and should be cited. There is no requirement to cite these works unless the editor has indicated otherwise*.**]**

**Response** : We carefully reviewed the reviewer comments, and we did not find any recommendation to cite specific previously published works.

- **Comment** 6: [ *Please review your reference list to ensure that it is complete and correct. If you have cited papers that have been retracted, please include the rationale for doing so in the manuscript text, or remove these references and replace them with relevant current references. Any changes to the reference list should be mentioned in the rebuttal letter that accompanies your revised manuscript.***]**

**Response** : We have carefully reviewed the reference list to ensure that it is complete and accurate. None of the cited works are retracted. Additionally, as suggested, we have incorporated four recent references from 2024 and 2025 to further strengthen the manuscript. Kindly refer to the revised version of the manuscript for these updates.

**Comments from Reviewer 1 and Responses**

- **Comment 1:** **[** *Summarize the abstract so that it covers the proposed algorithm in a simplified manner***.*]***

**Response :** Thank you for your suggestion. We have revised the abstract to provide a concise and simplified summary of the proposed algorithm, highlighting its key components and contributions. Please refer to the updated abstract in the revised manuscript.

**Comment 2: [***There is no need to refer to reference [9] twice in this paragraph, but to refer to it only once in the second location*: **]**

**Response :** Thank you for pointing this out. We have revised the manuscript to cite reference [10] (previously [9], due to the addition of a new reference) only once in the second location, as suggested. This change can be found in the revised manuscript on page 9, line 205.

**Comment 3:** [ *Add references published in the years 2024-2025, with at least two references for each year mentioned above.***]**

**Response :** We thank the reviewer for this valuable suggestion. In accordance with the recommendation, we have added four recent references [1-4] (two from 2024 and two from 2025) to strengthen the related work section and ensure the manuscript reflects the most up-to-date research..

- **Comment 4:** [*Showcase future work more broadly and clearly***]**

**Response :** We thank the reviewer for this suggestion. In response, we have expanded the future work. Please refer to revised manuscript on page 25, lines 572–577, for the revised text.

**References**

[1] Xie, J., Meng, Y., Zhao, Y., Nguyen, A., Yang, X., & Zheng, Y. (***2024***, March). Dynamic semantic-based spatial graph convolution network for skeleton-based human action recognition. In *Proceedings of the AAAI conference on artificial intelligence* (Vol. 38, No. 6, pp. 6225-6233)

[2] Jang, S., Lee, H., Kim, W. J., Lee, J., Woo, S., & Lee, S. (***2024***). Multi-scale structural graph convolutional network for skeleton-based action recognition. *IEEE Transactions on Circuits and Systems for Video Technology*, *34*(8), 7244-7258.

[3] Chen, D., Chen, M., Wu, P., Wu, M., Zhang, T., & Li, C. (***2025***). Two-stream spatio-temporal GCN-transformer networks for skeleton-based action recognition. *Scientific Reports*, *15*(1), 4982.‏.

[4] Zhang, Y., & Wang, Y. (2025). A comprehensive survey on RGB-D-based human action recognition: algorithms, datasets, and popular applications. *EURASIP Journal on Image and Video Processing*, ***2025***(1), 15
